# Effectiveness of empathy portfolios in developing professional identity formation in medical students: a randomized controlled trial

**DOI:** 10.1186/s12909-024-05529-5

**Published:** 2024-05-30

**Authors:** Munazza Baseer, Usman Mahboob, Neelofar Shaheen, Bushra Mehboob, Ayesha S Abdullah, Uzma Siddique

**Affiliations:** 1 Department of Health Professions Education and Research, Peshawar Medical College, Riphah International University, Peshawar, Pakistan; 2 Institute of Health Professions Education & Research, Khyber Medical University, Peshawar, Pakistan; 3 Department of Health Professions Education and Research, Peshawar Medical College, Riphah International University, Peshawar, Pakistan; 4 Department of Oral and Maxillofacial Surgery, Peshawar Dental College, Riphah International University, Peshawar, Pakistan; 5 Department of Health Professions Education and Research, Peshawar Medical College, Riphah International University, Peshawar, Pakistan

**Keywords:** Empathy, Medical students, Portfolios, Professional identity formation

## Abstract

**Background:**

Medical education requires innovative strategies to enhance empathic skills and the formation of professional identities among students. However, evidence-based teaching of empathy and professional identity formation is inadequately represented, particularly in medical curricula. This study investigated the effectiveness of empathy portfolios in developing Professional Identity Formation (PIF) among medical students and the correlation between empathy and PIF. The objectives of this study were to determine the effectiveness of empathy portfolios for teaching and nurturing PIF in medical students and to investigate the correlation between empathy and PIF.

**Methods:**

A randomized controlled trial was conducted at Peshawar Medical College, Pakistan. The protocol adhered to CONSORT guidelines. A total of 120 students participated in the study. Empathy and PIF were assessed using two validated questionnaires JSPE-S and PIQ before randomization. The participants were randomized in a stratified fashion into the experimental (*n* = 60) and control (*n* = 60) groups. The Participants in the intervention group attended a training workshop on portfolio use. Students maintained their portfolios and wrote reflections on incidents that evoked empathy. Independent t-tests were performed to determine whether the control and experimental groups differed in terms of mean empathy and PIF scores, and Pearson’s correlation analyses were used to investigate the relationships between pre- and post-empathy, and pre-post-PIF.

**Results:**

The mean post-test scores on the Empathy and PIF showed a statistically insignificant difference of 0.75 +-17.6 for empathy and 0.45 ± 8.36 for PIF. The intervention had little influence on empathy and PIF scores, as evidenced by nonsignificant effect sizes of 0.32 and 0.36 for empathy and PIF respectively.A strong positive correlation was found between Pre-Empathy and the PIF-Total score (0.519), and between Post- empathy and the PIF-Total score (0.395) (*p* < 0.001).

**Conclusions:**

Empathy had a positive linear correlation with PIF; however, the use of empathy portfolios as a three-week single-point intervention was ineffective at nurturing PIF.

**Supplementary Information:**

The online version contains supplementary material available at 10.1186/s12909-024-05529-5.

## Background

One of the goals of medical education is to assist students in developing a professional identity that is well-formed and mature [1, 2]. In recent years, professional identity formation (PIF) has received much attention in the medical education literature since it establishes the moral foundation for one’s medical practice by ensuring that everyone has acquired a professional identity to “think, act, and feel like a physician” [3–5]. PIF is a complex process involving internalising professional values, roles, and behaviours and shaping how healthcare professionals perceive themselves and their interactions with patients and colleagues [5]. However, medical schools have struggled to develop effective strategies to promote PIF [6]. Professional identity development in medical students can be impeded by factors such as bias, disregard for humanistic elements, and the process of dehumanization [6].

When considering what qualities make a good physician, the literature and related research support the importance of cognitive aspects such as knowledge and professionalism, and emotional elements such as empathy and compassion [7]. Empathy is a valuable attribute in the medical field since it plays a critical role in building a solid and meaningful relationship between physicians and their patients [8]. Several studies have reported decreased empathy levels in the later stages of medical professionals’ training programs [9]. Multiple factors contribute to this decline; doctors prioritize medical skills over humanistic understanding owing to high patient loads and a tendency to create distance from patients [10].

Despite its significance, insufficient consideration has been given to the role of empathy in developing students’ professional identity. The absence of empathy in the existing models of professional identity requires further probing [11].

Medical education struggles to promote empathy due to workload, emotional exhaustion, desensitization, and hidden curriculum [12, 13]. Zhou et al. [14] suggested employing portfolios for nurturing empathy and recommended exploring empathy’s potential link with PIF. The purpose of this study is to investigate the effectiveness of empathy portfolios in developing PIF among medical students. In particular, it investigates if there is a correlation between empathy and PIF and evaluate the impact of empathy portfolios on the development of students’ professional identity. Given this context, our study aimed to answer the following research questions:

Would Empathy portfolios prove to be an effective intervention for nurturing medical students’ professional identity formation?

Is there a correlation between empathy and PIF among medical students?

## Methods

**Fig. 1:**
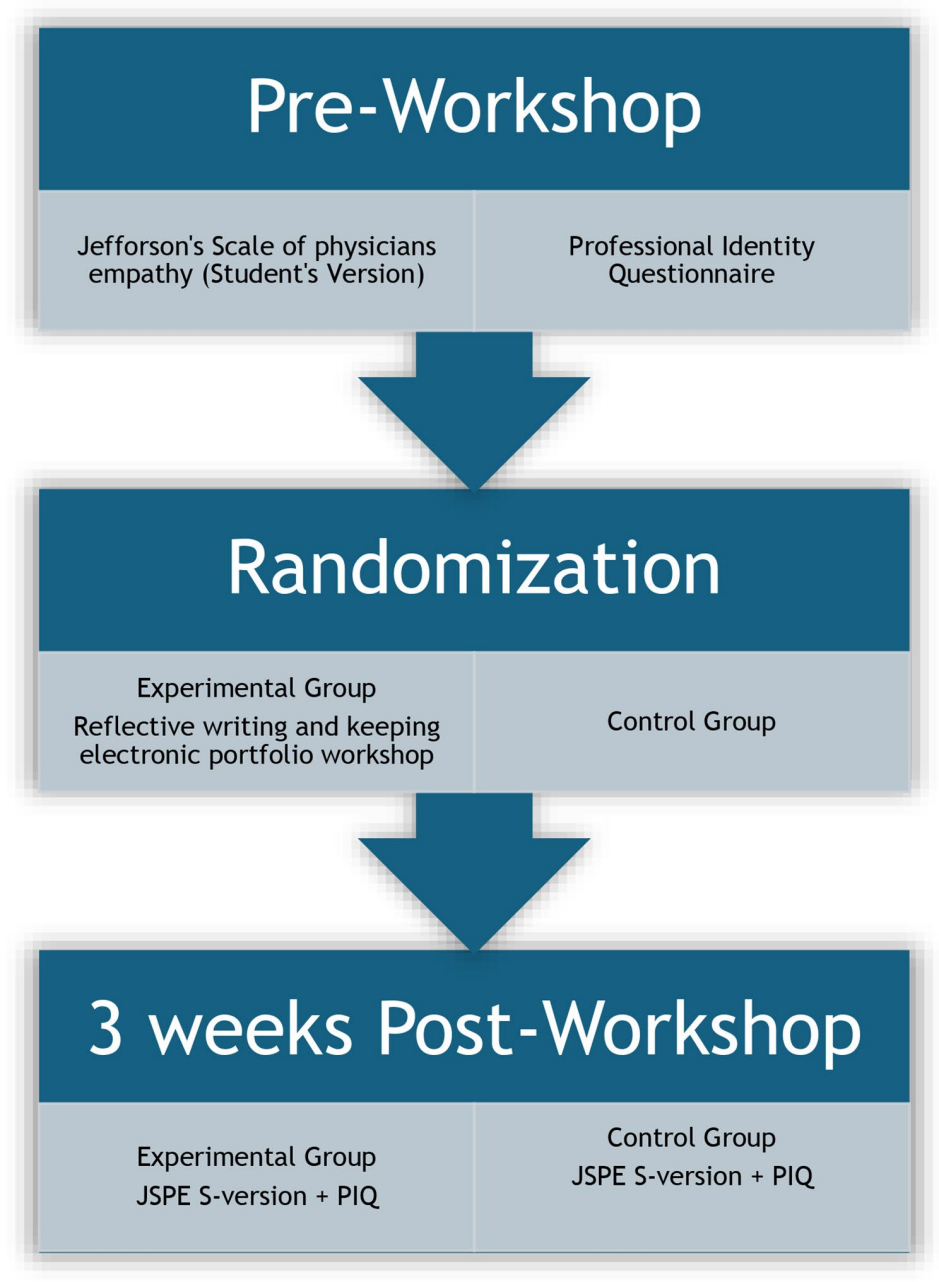
Study design: Effectiveness of empathy portfolios in developing PIF in undergraduate medical students

The study duration ranged from April 2023 to September 2023. A randomized controlled trial (Fig. 1) was designed and conducted at Peshawar Medical College, Peshawar, Pakistan. The intervention primarily involved the use of major hospitals attached to the medical college which served as the educational environment for developing PIF for the students who participated in this educational trial. These hospitals provide a clinical environment where medical students interact with patients, allowing them to apply and refine their PIF. The consolidated standards of reporting trials (CONSORT) guidelines for Randomized Control Trials were followed to ensure the quality of the study [15]. Our study is an educational intervention that does not involve any patients or health outcomes and does not fall under the criteria of a clinical trial as defined by the International Committee of Medical Journals Editors (ICMJE) [16] Therefore, we did not register it in any clinical trial registry.

### Ethical approval

was obtained from the Advanced Study Research Board, Khyber Medical University Peshawar, approval number DIR/KMU-AS&RB/EE/002050 meeting held on 31/05/2023, and the Institutional Review Board, Peshawar Medical College, Peshawar, approval number PRIME/IRB/2023 − 532, meeting held on 31/05/2023.

### Sample size and sampling technique

A total of 120 third year MBBS students who had spent at least three months in a hospital setting (clinical clerkships) were included in the study. Stratified random sampling was employed to draw a sample with a probability proportional to the size of the stratum (male and female students) using Open Epi. With a 95% two-sided confidence interval, the sample size for the control and experimental groups was calculated as 60 for each group. A pre-test was taken as a baseline for empathy, using the.

***Jefferson Scale of Physician Empathy (JSPE) for Medical Students*** and ***Professional Identity Questionnaire*** (PIQ) for PIF [17, 18].

Clinical rotations, normally started in the third year of medical school are a critical shift from theoretical knowledge gained in the classroom to real-world application in healthcare settings. Therefore, during this time, when students are navigating the complexities of patient care for the first time, interventions aimed at developing professional identity, like empathy portfolios, may have a significant impact. Third-year medical students grapple with issues of professionalism, empathy, and their changing role as healthcare providers as they engage more towards the clinical environment. At this point, interventions aimed at helping them develop their professional identities can help set the groundwork for their future practice. Even though third-year students might not have much experience in clinical settings, their involvement in the research offers a chance to evaluate the initial impacts of interventions on the development of professional identities.

### Control Group

The control group included 60 medical students who did not maintain their empathy portfolio during the study period. Instead, they followed the traditional routine curriculum without specific empathy-focused activities.

### Experimental/trial group

The experimental group consisted of 60 students who were exposed to an educational intervention that included empathy triggers and reflective practices. Participants were introduced to empathy portfolios and given comprehensive instructions on how to use and maintain them effectively. Participants signed the confidentiality agreements as they were informed about the necessity of respecting group boundaries and confidentiality, emphasising the potential impact on the intervention’s success. The randomly selected students were exposed to clinical rotations for the first time, but consideration of empathy triggers and knowledge of PIF was never part of their curriculum. For this reason, previous clinical rotations might have minimally influenced the PIF of participants as a confounding variable.

### Data collection procedure

Sixty students of the experimental group participated in a 2.5-hour training workshop where they learned how to create electronic portfolios on Google sites and document guided reflections on experiences that made them feel empathetic. Participants were trained by two experienced facilitators by demonstrating examples of how to collect artefacts (picture/ video/ audio) of any interaction or events that made them feel empathetic and reflect on them. After the workshop, participants were required to maintain their portfolios and record their interactions with patients, colleagues, and healthcare professionals for a duration of three weeks. During post-clinical hours, participants used their user-specific login credentials to access their electronic portfolios and compose guided reflections. Although the risk of potential data contamination was there, user-specific log-ins, along with supervised monitoring and a confidential intervention space served as measures to minimize the risk. Following three weeks of portfolio engagement, the participants again filled out Jefferson’s Scale for Empathy and the PIF Questionnaire post-test using Google Forms.

### Instrumentation

#### Professional identity questionnaire

Brown et al. created the PIQ (Professional Identity Questionnaire) to measure an individual’s level of social affiliation with a given group. It has been used to assess the level of professional identity among nurses working in several South English hospitals. The questionnaire has ten items that are scored on a 5-point Likert scale from 1 (never) to 5 (often). To guarantee uniformity, items F through J are negatively written, and their scoring is inverted. The PIQ gives a quantitative measure of professional identity by producing a total score. The PIQ has been validated by Daan et al. (2021) as a quantitative measure of professional identity formation among medical students in medical educational settings. Internal consistency analysis showed a Cronbach’s alpha value of 0.82.

### Jefferson scale of empathy medical student version (JSPE -Sversion)

The JSPE questionnaire uses a self-report style to test empathy levels. It consists of 20 items scored on a 7-point Likert scale, with 1 being strongly disagree and 7 being strongly agree. The JSPE has a score range of 20 to 140, with higher scores suggesting stronger levels of empathy. The JSPE-S version has 10 items that are positively phrased and pertain to “perspective taking,” whereas the other 10 items are negatively worded and pertain to topics such as “compassionate care” and “standing in the patient’s shoes.” On a Likert scale of 7 to 1, the negatively phrased items were reverse rated. It is worth noting that the JSE-MS was not translated in this study because all participants were fluent in English and had acquired their education in English.

### Data analysis

Quantitative data collected from both the pre-test and post-test questionnaires were compiled and organized for analysis. Independent t-tests were performed to determine whether the control and experimental groups differed in terms of mean empathy and PIF scores; paired sample t-tests were used for pre- and post-empathy; and pre- and post-PIF. Pearson’s correlation analyses were used to investigate relationships between pre- and post-empathy and pre- and post-PIF scores. Cohen’s d effect sizes were calculated to measure the effect of intervention.

## Results

Participants in the study comprised 120 third year MBBS students. 62.5% (*n* = 75) of them were male, and 37.5% (*n* = 45) were female. The students who took part in the study ranged in age from 20 to 22 years old, with an average age of 21 years.

The intervention had little influence on empathy and PIF scores, as evidenced by the minor differences and nonsignificant effect sizes (0.32 and 0.36 for empathy and PIF respectively). The study was unable to find statistically significant differences in post-test scores for PIF or empathy between the experimental and control groups or between participants, regardless of gender, as shown in Table 1.

**Table 1:** Independent t-tests of post-test scores in the control and experimental groups

Post-tests	Group	Mean	SD	t-value	*p*-value
Post Empathy	Control	83.10	12.590	-1.793	0.75
	Experimental	88.12	17.637		
	Male *n* = 75	84.35	15.305	-1.155	0.250
	Female *n* = 45	87.71	15.672		
Post PIF	Control	28.87	5.401	-2.023	0.45
	Experimental	31.47	8.366		
	Male *n* = 75	30.11	7.061	− 0.118	0.906
	Female *n* = 45	30.27	7.328		

The Pre-Empathy-Total score showed a strong positive correlation with the Pre-PIF-Total score (0.519), and this correlation was statistically significant (*p* < 0.001). Post-PIF-Total showed a strong positive correlation with Post-Empathy-Total (0.395), and this correlation was statistically significant (*p* < 0.001). Table 2 is given below.

**Table 2:** Pearson Correlations between Pre- and Post-empathy and PIF (*n* = 120)

		Pre-PIF	Pre-empathy	Post empathy	Post PIF
Pre-PIF	r	1			
Pre-Empathy	r	0.519**	1		
Post-Empathy	r	− 0.129	− 0.082	1	
Post-PIF	r	− 0.009	0.109	0.395**	1

**. Correlation is significant at the 0.01 level (2-tailed)

## Discussion

The current study is the first experimental study to examine the efficacy of an intervention promoting professional identity through empathy portfolios during medical school. One possible explanation for these nonsignificant results could be related to the complexity of Professional Identity Formation. According to Sarraf-Yazdi et al. [19]. , professional identity in the medical field is influenced by many factors, including personal values, experiences, and role modelling by established professionals. Although a well-intentioned intervention was used, the empathy portfolios might not have adequately addressed all these factors. Moreover, the duration of the intervention may not have been long enough to manifest substantial changes. Research by Fathi et al. (2023) indicated that interventions spanning a more extended period tend to yield more profound effects on professional identity [20].

The results of this study on the effectiveness of empathy portfolios in developing PIF among medical students offer several important insights. This study revealed slight improvements in the measured variables (empathy and PIF) both before and after the intervention. The results of this study showed that the structured empathy portfolio and routine hospital clerkships did not differ significantly from each other in the development of empathy and PIF. However, the experimental group performed slightly better on empathy and PIF than the control group. Since PIF is a more complex process, a 3-week period may not have been sufficient to demonstrate any significant difference; however, this study provides insight into whether this approach is sufficient to observe any change in empathy or PIF. Furthermore, the nonsignificant differences between the control and experimental groups underscore the importance of considering diverse teaching methods. According to Hookmani [21], combining empathy training with interactive workshops, reflective practices, and mentorship programs has shown promising results in enhancing PIF. Thus, future interventions might benefit from a multifaceted approach that incorporates various teaching strategies and integrates empathy development into the broader context of medical professionalism and PIF. The lack of gender differences in the outcomes aligns with previous research by Oosterhoff & Yunus [22], suggesting that gender does not significantly influence the development of empathy or professional identity in medical students. Regarding the second research question, the pre-PIF total score strongly correlated with the pre-empathy total score and post-empathy post-PIF score.

The study by Artioli et al. [23] showed that interventions incorporating reflective practices, such as journaling and debriefing sessions, can lead to profound self-awareness, skills development, professional growth, empathic attitudes and sensitivity towards emotions.

Given the dynamic nature of identity and the significant impact of informal interactions outside the classroom on PIF among medical students, educational institutions must offer sufficient network and institutional support to develop PIF [24]. This can be achieved by facilitating casual interactions in diverse learning environments to promote successful transformations.

This study has significant implications for medical education and the professional growth of medical students. The process of self-regulation, known as PIF, is characterized by ongoing and deliberate efforts to manage and influence internal and external influences [25]. This process necessitates a certain level of motivation to effectively govern the conversation between these factors. Hence, medical schools should have student-centred and individualized learning experiences augmented by reflection. These measures are essential for fostering motivation and facilitating a positive PIF process. Furthermore, medical students need to consider their professional trajectory and current stage of development. Therefore, using a questionnaire to quantify PIF, as implemented in this research, could be a valuable tool for evaluating PIF among medical students.

## Conclusions

In summary, although empathy portfolios did not demonstrate significant effectiveness in our study, their investigation provided valuable insights into the complex process of professional identity formation in medical education. By using innovative teaching methods and continually developing educational interventions, educators can better prepare medical students for the challenges and responsibilities of modern healthcare practice.

### Strengths and limitations

This study forges the use of empathy portfolios as a tool to help develop Professional Identity Formation (PIF) among medical students. The strength of the study lies in its RCT design, enhancing the credibility of the findings, deepening understanding of the interplay between the constructs, and potentially informing future educational interventions. While the study demonstrates a positive effect of the intervention, the effect size is small. The study’s timeframe of three weeks might underestimate the full potential of the effect of empathy portfolios on PIF as portfolios are typically intended for longer-term use. Conducting the study within a single institution limits the generalizability of the findings. Replicating the study across multiple institutions would enhance the validity of the results. Various measures were adopted to minimize contamination between groups, but the inherent risk of data contamination in a single-institution study remains.

### Future directions

Further studies might examine whether more prolonged exposure to portfolios, as recommended, may result in greater effect sizes and whether sustained effects could be observed over an extended period. Future research might also explore the potential difficulties and impediments to successfully integrating empathy portfolios into the medical curriculum.

### Electronic supplementary material

Below is the link to the electronic supplementary material.

Supplementary Material 1

## Data Availability

The datasets generated in response to filling the questionnaire are available from the corresponding author on request.
