# Supplementary material for: Effectiveness of empathy portfolios in developing professional identity formation in medical students: a randomized controlled trial
Source: BMC Med Educ. 2024 May 30;24:600. doi: 10.1186/s12909-024-05529-5 (PMC11140931; doi:10.1186/s12909-024-05529-5)

**Data collection Instrument**

***Jefferson Scale of Physician Empathy (JSPE) For Medical Students***

The JSPE questionnaire uses a self-report style to test empathy levels. It consists of 20 items scored on a 7-point Likert scale, with one being strongly disagree and seven being strongly agree. The JSPE has a score range of 20 to 140, with higher scores suggesting stronger levels of empathy. The JSPE-S version has ten items that are positively phrased and pertain to "perspective taking. " In contrast, the other ten items are negatively worded and pertain to topics such as "compassionate care" and "standing in the patient's shoes." The negatively phrased items were reverse-rated on a Likert scale of 7 to 1. It is worth noting that the JSPE-S was not translated in this study because all participants were fluent in English and had acquired their education in English. This scale encompasses 20 Likert-type items, rated from 1 (strongly disagree) to 7 (strongly agree), leading to a summed score ranging from 20 to 140, with a higher score indicating higher levels of empathy. A validated Professional Identity Questionnaire (PIQ) was used to measure the students' professional identity (Daan et al., 2021).

**Questionnaire #1 :**

**Jefferson Empathy Scale For Medical Students**

**Strongly disagree 1**

**Moderately disagree 2**

**Slightly disagree 3**

**Neutral 4**

**Slightly agree 5**

**Moderately agree 6**

**Strongly agree 7**

1. Physicians’ understanding of their patients’ feelings does not influence medical or surgical treatment

2. Patients feel better when their physicians understand their feelings.

3. It is difficult for a physician to view things from patients’ perspectives.

4. Understanding body language is as important as verbal communication in the physician–patient relationship.

5. A physician’s sense of humor contributes to a better clinical outcome.

6. Because people are different, it is difficult to see things from patients’ perspectives.

7. Attention to patients’ emotions is not important in history taking.

8. Attentiveness to patients’ experiences does not influence treatment outcomes.

9. Physicians should try to stand in their patients’ shoes when providing care for them.

10. Patients value a physician’s understanding of their feelings, which is therapeutic in its own right.

11. Patients’ illnesses can be cured by medical or surgical treatment.

12. Asking patients about what is happening in their personal lives is not helpful.

13. Physicians should understand what is going on by paying attention to nonverbal cues and body language.

14. I believe that emotion has no place in the treatment of medical illness.

15. Empathy is a therapeutic skill without which the physician’s success is limited.

16. Physicians’ understanding of the emotional status of patients, as well as that of their families, is important.

17. Physicians should try to think like their patients in order to render better care

18. Physicians should not allow themselves to be influenced by strong personal bonds with patients.

19. I do not enjoy reading nonmedical literature or the arts.

20. I believe that empathy is an important therapeutic factor in medical treatment.

**Professional Identity Questionnaire**

***Professional identity Questionnaire***

Brown et al. created the PIQ (Professional Identity Questionnaire) to measure an individual's level of social affiliation with a given group. It has been used to assess the level of professional identity among nurses working in several South English hospitals. The questionnaire has ten items scored on a 5-point Likert scale from 1 (never) to 5 (often). To guarantee uniformity, items F through J are negatively written, and their scoring is inverted. The PIQ gives a quantitative measure of professional identity by producing a total score. Previous research has found that the PIQ has a satisfactory dependability (Cronbach's = 0.77).

**Questionnaire # 2**

**Please indicate on scale of 1 to 5 how much the given statements are true for you.**

**Never                Seldom Sometimes              Often Very Often**

**1                         2       3 4                     5**

1. I am a person who considers the doctors’ group important. 1 – 2 – 3 – 4 - 5
2. I am a person who identifies with the doctors’ group. 1 – 2 – 3 – 4 - 5
3. I am a person who feels strong ties with the doctors’ group. 1 – 2 – 3 – 4 - 5
4. I am a person who is glad to belong to the doctors’ group. 1 – 2 – 3 – 4 - 5
5. I am a person who sees myself belonging to the doctors’ group. 1 – 2 – 3 – 4 - 5
6. I am a person who makes excuses for belonging to the doctors’ group. 1 – 2 – 3 – 4 - 5
7. I am a person who tries to hide belonging to the doctors’ group. 1 – 2 – 3 – 4 - 5
8. I am a person who feels held back by the doctors’ group. 1 – 2 – 3 – 4 - 5
9. I am a person who is annoyed to say that I’m a member of

the doctors’ group. 1 – 2 – 3 – 4 - 5

1. I am a person who criticizes the doctors’ group. 1 – 2 – 3 – 4 – 5

**Empathy Portfolio Reflective Prompts**

**Reflective Prompts**

**What happened? (Describe the significant event and the context for it in few lines) that made you feel empathy for someone(patient)?**

**What have you learned from the incidence? (Identify opportunities for learning and actions to be taken forward)**

**What role a doctor can play to make this situation better?**

**Would this incident will bring any change to your medical practice in the future? If yes, how?**

**Sample Size calculation**


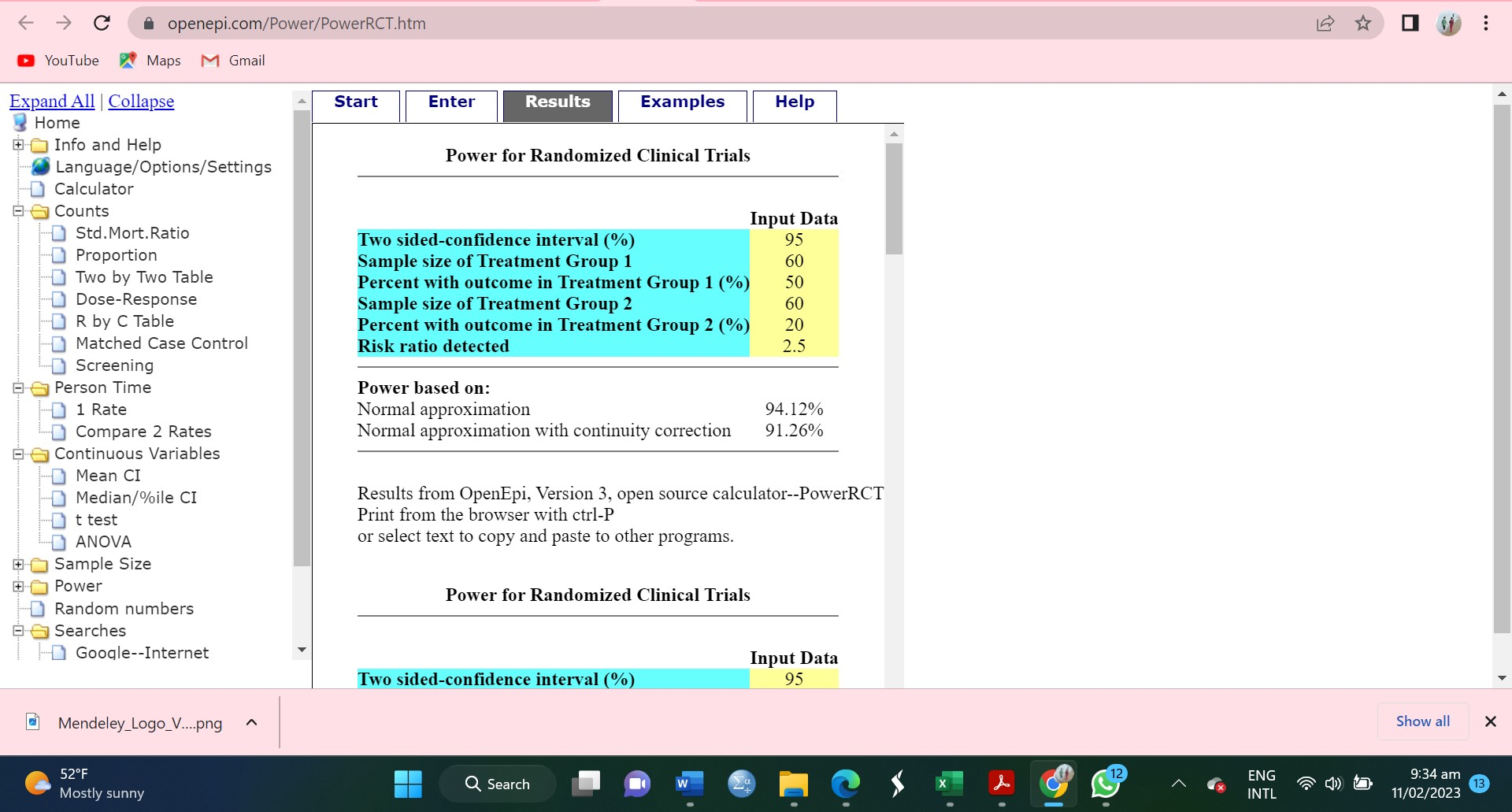

Supplement: Supplementary file 1 — Supplementary Material 1 [file 12909_2024_5529_MOESM1_ESM.docx]
